# Immune checkpoint blockade lowers the threshold of naïve T-cell priming to drug-associated antigens in a dose-dependent fashion

**DOI:** 10.1093/toxsci/kfae118

**Published:** 2024-09-17

**Authors:** Sophie Grice, Katy Saide, Liam Farrell, Georgia Wells, Catherine Betts, Sean Hammond, Dean J Naisbitt

**Affiliations:** Centre for Drug Safety Science, Department of Molecular and Clinical Pharmacology, University of Liverpool, Liverpool L69 3GE, United Kingdom; Clinical Pharmacology and Safety Science, AstraZeneca, Cambridge, United Kingdom; Centre for Drug Safety Science, Department of Molecular and Clinical Pharmacology, University of Liverpool, Liverpool L69 3GE, United Kingdom; Centre for Drug Safety Science, Department of Molecular and Clinical Pharmacology, University of Liverpool, Liverpool L69 3GE, United Kingdom; Clinical Pharmacology and Safety Science, AstraZeneca, Cambridge, United Kingdom; ApconiX, Alderley Edge SK10 4TG, United Kingdom; Centre for Drug Safety Science, Department of Molecular and Clinical Pharmacology, University of Liverpool, Liverpool L69 3GE, United Kingdom

**Keywords:** adverse drug reaction, T-lymphocytes, immunogenicity, immune dysregulation

## Abstract

A growing body of clinical and experimental evidence indicates that immune checkpoint blockade enhances patient susceptibility to hypersensitivity reactions to co-administered medications. In this study, we utilized an *in vitro* T-cell priming assay to demonstrate one of the mechanistic hypotheses on how this occurs; through lowering of the threshold in patients to elicit aberrant T-cell responses. We outline the dependency of *de novo* T-cell priming responses to drug-associated antigens on dose at initial exposure. Findings support the aforementioned hypothesis and offer an experimental representation of fundamental parameters at play in hypersensitivity reactions to low molecular weight compounds.

Immune checkpoint inhibitor (ICI) therapy is an efficacious anticancer therapy that modulates patient’s immune response to tumors. To date, the predominant therapeutic strategy has been to administer antagonistic monoclonal antibodies specific for immune checkpoints and their ligands such as programmed cell death 1 (PD-1), programmed cell death ligand 1 (PD-L1), and cytotoxic T lymphocyte antigen 4 (CTLA-4) in monotherapy or in combination with other anticancer agents. These drugs have proven highly efficacious treatments for a variety of cancer indications such as melanoma and non-small cell lung cancer (Hoos 2016). This therapeutic success has; however, been accompanied by association with immune-related adverse events (irAEs). The majority of these irAEs are the result of the intended pharmacology of the drug (elicitatory immunomodulation) and as such they are often referred to as on-target, off-tissue toxicities. It is generally believed that many of these irAEs represent the targeting of self-antigens, and therefore represent an iatrogenic autoimmune disease. This dysregulation of immunological perception also appears to extend to xenobiotic-derived antigens, and an emerging clinical issue is the observation of an increased incidence of hypersensitivity reactions to co-administered drugs (Hammond et al. 2022a, 2022b). Previously, we have investigated clinically defined hypersensitivity reactions to sulfasalazine and amidotrizoate (Ford et al. 2018; Hammond et al. 2021; Hammond et al. 2022b) and experimentally demonstrated a plausible mechanism by which the checkpoint inhibitor may have led to the deleterious clinical outcome. A further drug this phenomenon has been observed with is sulfamethoxazole (Urasaki et al. 2021), in this case, our own mechanistic studies preceded the clinical observations (Gibson et al. 2014, 2017). In the case of both sulfa drugs sulfasalazine and sulfamethoxazole, the hapten-forming reactive metabolite which gives rise to neoantigenic determinants (responsible for antigens responsible for activating some but not all of the pathogenic T-cells)—see Pharmacological interaction and hapten mechanisms outlined in the following reviews (Naisbitt et al. 2003; Pichler et al. 2011). This facilitated an initial characterization of checkpoint inhibitor-mediated augmentation of T-cell priming responses (Gibson et al. 2014, 2017; Hammond et al. 2022b). While this augmentation of responses is established, the thresholds that apply to T-cell priming to these drug-associated antigens in the context of checkpoint inhibition were yet to be explored.

Herein we conducted a series of *in vitro* T-cell priming assays on healthy derived primary cells to explore the dose-dependence of *de novo* T-cell responses to the reactive metabolite nitroso sulfamethoxazole (SMX-NO). We compared how the dose-response relationships within these assays behave in the presence and absence of *in vitro* checkpoint-blocking antibodies. The findings we present address limitations of previous mechanistic studies through the characterization of the dose-response relationship of the T-cell priming response and illustrate clear intra-individual differences in terms of the threshold dose required to elicit a response. Taken together, this offers a clear experimental depiction of how ICIs reduce the threshold for drug-specific T-cell priming across a population, leading to an increased number of individuals susceptible to hypersensitivity reactions.

Peripheral blood mononuclear cells (PBMC) were isolated as outlined in Ogese et al. (2020) from healthy volunteer leukocyte cones purchased from the National Health Service Blood and Transplant (NHSBT). CD14+ cells and CD3+ cells were isolated via MACS separation and used in a co-culture of matured monocyte-derived dendritic cells and CD3+ T-cells with and without anti-PD-L1 mAb (5 µg/ml) and SMX-NO (1 to 60 µM) incubated for 10 to 12 d. The optimum priming concertation for SMX-NO (without checkpoint inhibition) was selected as the highest study concentration (Gibson et al. 2014). This was then reduced to a concentration of SMX-NO where priming was not observed without checkpoint inhibition. T-cell responses with and without checkpoint inhibition were then compared. The physiological concentration of SMX-NO is difficult to define; however, its precursor SMX hydroxylamine is detected in patient plasma at low µM concentrations (Gill et al. 1996). The anti-PD-L1 antibody was used at the manufacturer’s indicated concentration. Re-challenge with media control or SMX-NO was carried out with the addition of a second set of matured monocyte-derived dendritic cells and proliferation and cytokine secretion were measured 2 d later (Fig. 1).

**Fig. 1.**
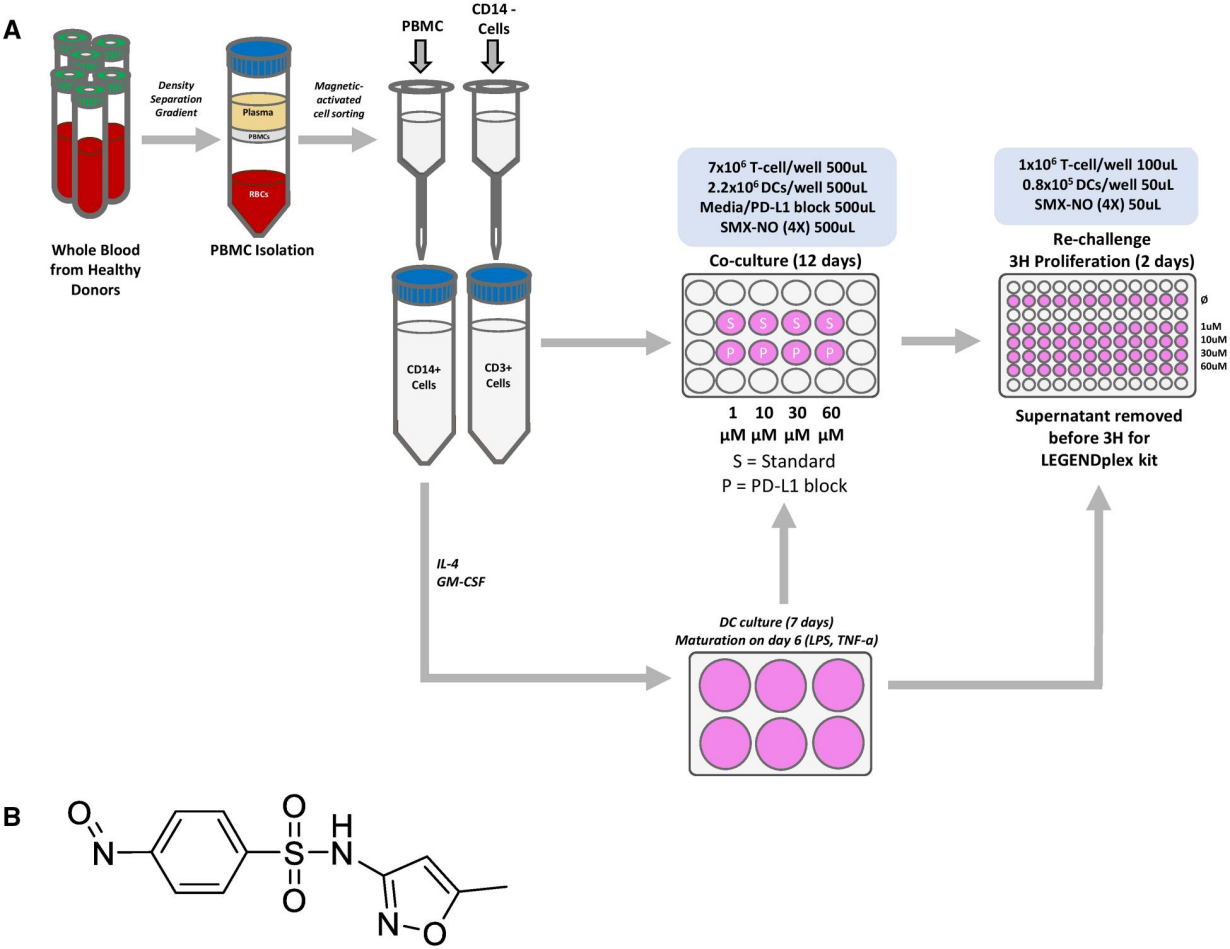
A) Schematic representation of T-cell priming assay. PBMC (peripheral blood mononuclear cells) were isolated by density centrifugation. CD14 and CD3 cells were separated in line with manufacturer’s instructions (Miltenyi). Monocyte-derived dendritic cells were cultured as outlined in Ogese et al. (2020). Matured autologous monocyte-derived dendritic cells (2.3 × 10^5^ cells/mL) were harvested and co-cultured with CD3+ T-cells (7 × 10^6^ cells/mL). Programmed cell death ligand 1 (PD-L1) monoclonal antibodies (BioLegend) were added at a final concentration of 5 µg/mL for 1 h then SMX-NO (1 to 60 µM) was added. The co-culture was incubated (95% air, 5% CO_2_, 37°C, humidified) for 10 to 12 d during this time the priming of naïve T-cells may occur resulting in the expansion of drug-specific T-cells. After incubation and washing cells were plated at (1 × 10^6^ cells/mL) and re-challenged with a second set of matured dendritic cells (1.6 × 10^5^ cells/mL) and either R9 medium as the negative control or SMX-NO (1 to 60 µM). After 48 h, supernatant was removed for cytokine analysis using cytometric bead-based immunoassays and cells were pulsed with 0.5µCi [3H]-thymidine per well during the final 16 h of incubation, proliferation was measured via incorporation of radioactivity. The stimulation index (SI) was calculated to determine whether drug-specific T-cells were formed and whether immune checkpoint inhibition (ICI) increases their production. An SI of 1.5 is indicative as a positive response where T-cell had been successfully primed. B) Structure of sulfamethoxazole nitroso (SMX-NO).

Under standard priming conditions (No ICI block) there is a dose-dependency to both the initial co-culture priming conditions and the re-challenge concentrations. Under standard conditions when *in vitro* priming concentrations of 10 µM for donor 3 (Fig. 2C), or 30 µM for donor 1 (Fig. 2A), and of 60 µM for donor 2 (Fig. 2B) were reached, T-cell priming occurred and T-cells responded by statistically significant proliferation (Fig. 2). There is inter-individual variation in optimal initial in-vitro priming concentration between donors, which may be due to altered PD-L1 expression on donor dendritic cells. Upon re-challenge with SMX-NO (1 to 60 µM) there is a dose-dependent increase in proliferation as re-challenge concentration of SMX-NO is increased (Fig. 2).

**Fig. 2.**
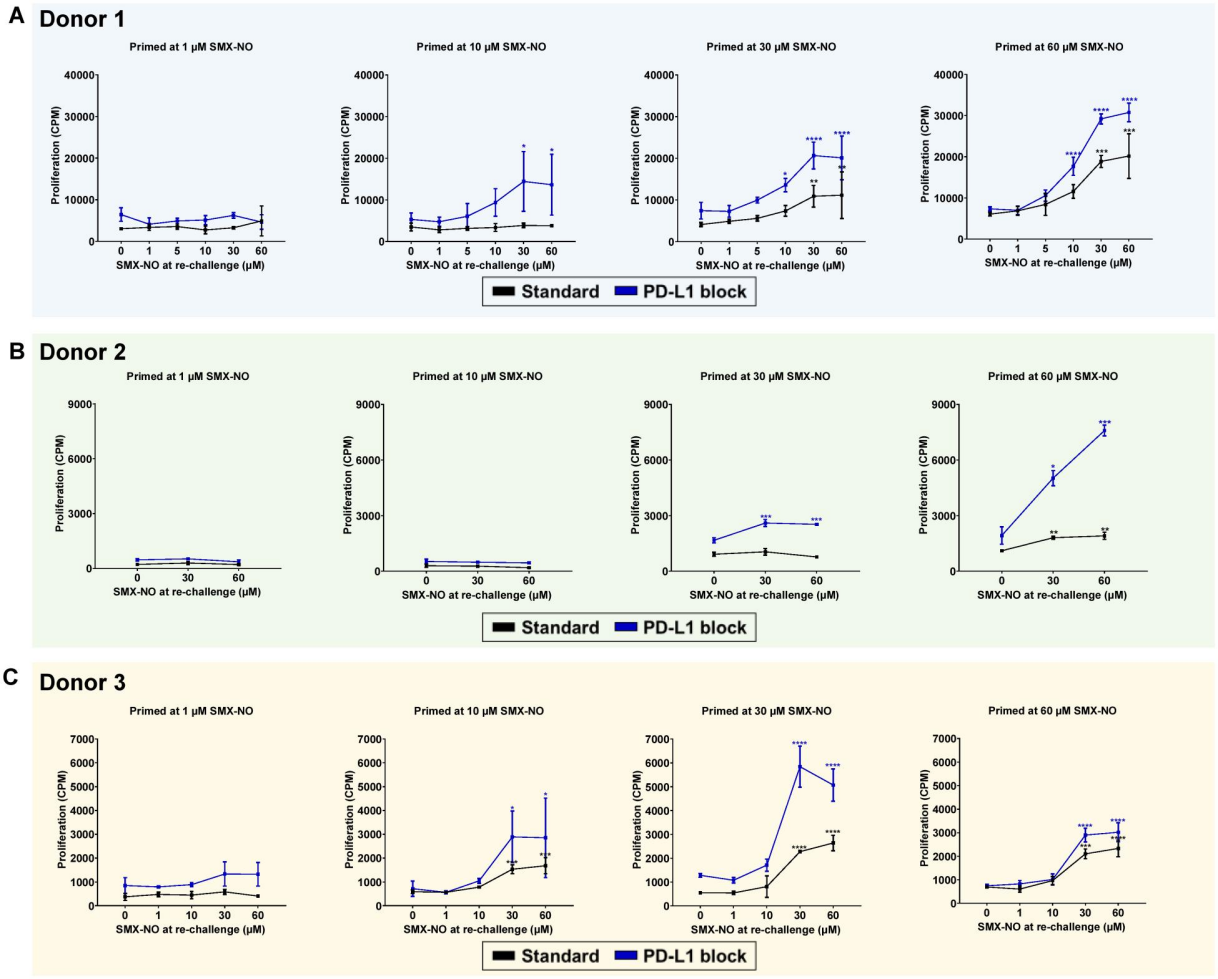
Assessment of immune checkpoint blockade on *de novo* T cell priming responses to the model antigen nitroso sulfamethoxazole (SMX-NO) in 3 healthy donors: A) Donor 1, B) Donor 2, and C) Donor 3. Data presented as mean counts per minute ± SD. Statistical significance compared to media-treated control using 2-way ANOVA; *P < 0.05, **P < 0.01, ***P < 0.001, and ****P < 0.0001.

FACS analysis of the supernatant revealed under standard priming conditions the secretion of IL-10 (*n* = 1) after initial priming at 10 µM SMX-NO, secretion of IL-10 and IL-5 (*n* = 1) and IL-13 (*n* = 2) after initial priming at 30 µM SMX-NO, and after initial priming concentration of 60 µM SMX-NO secretion of IL-5 and IL-13 (*n* = 2) and IL-10 (*n* = 1) was detected above background levels (Fig. 3A). Under standard conditions dose-dependent secretion of IFN-γ, IL-5, and IL-13 were observed for donor 1 with initial priming concentrations of 30 to 60 µM SMX-NO (Fig. 3B).

**Fig. 3.**
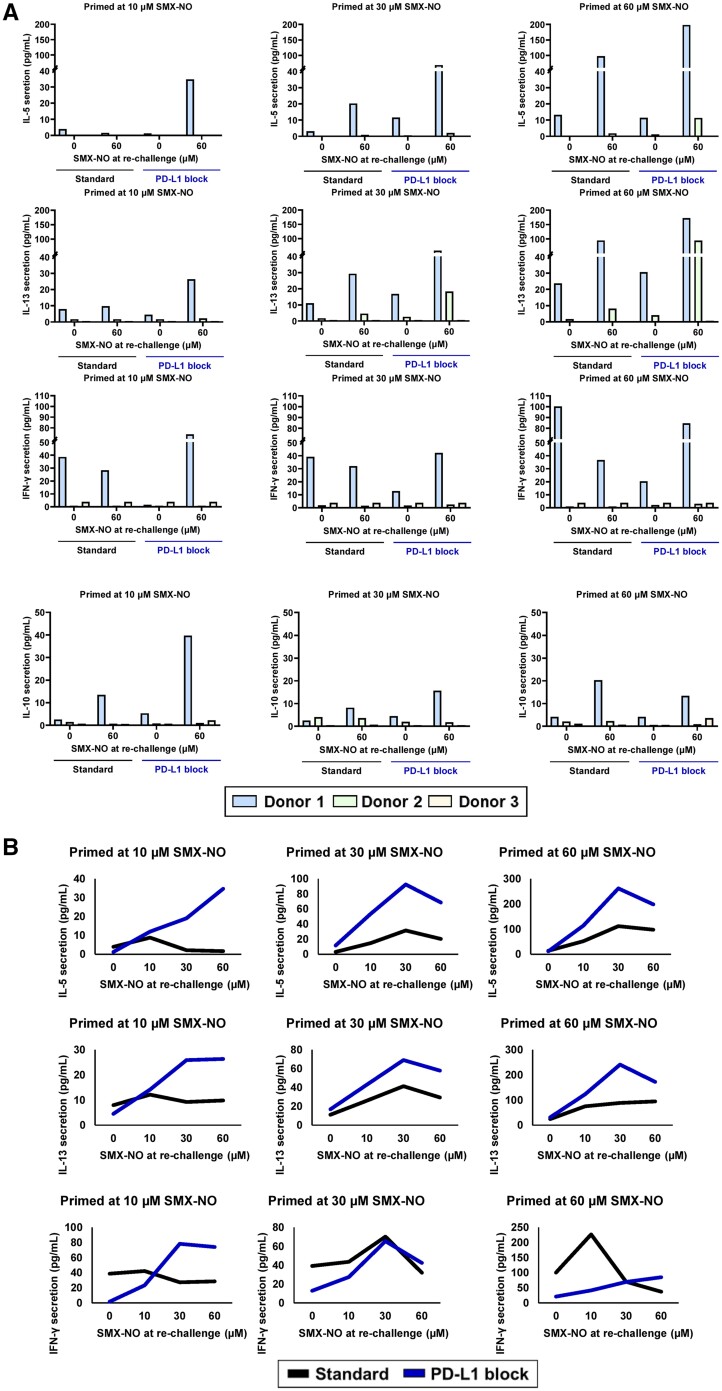
Assessment of immune checkpoint blockade on the secretion of cytokines after *de novo* T cell priming responses to the model antigen nitroso sulfamethoxazole (SMX-NO). Cytokines were quantified in T-cell-dendritic cell co-culture supernatant (25 μL) using bead-based immunoassays according to the manufacturer’s instructions (LEGENDplex, BioLegend Custom Human 11-plex panel). Fluorescent signals were measured using BD FACSCanto II. A) IL-5, IL-13. IFN-γ and IL-10 secretion from donors 1, 2, and 3 after re-challenge with media control or SMX-NO (60 µM) under standard or programmed cell death ligand 1 (PD-L1) priming conditions. B) Dose-dependent secretion of IL-5, IL-13, and IFN-γ from donor 1 after re-challenge with media control or SMX-NO (1 to 60 µM) under standard or PD-L1 block priming conditions.

Under “sub-optimal” initial co-culture priming concentrations for SMX-NO at 1 µM for donor 3 (Fig. 2C), at 10 µM for donor 1 (Fig. 2A), and 30 µM for donor 2 (Fig. 2B) T-cell priming was only observed with *in vitro* PD-L1 blockade, without immune checkpoint blockade T-cell priming could not occur (Fig. 2). This indicated that checkpoint inhibition resulted in a leftward shift of the dose-response curve for T-cell priming responses. At priming concentrations of 60 µM SMX-NO T-cell priming was observed regardless of immune checkpoint blockade for all donors, however, T-cell priming responses were greater with immune checkpoint blockade displayed by increased dose-dependent proliferation and cytokine secretion upon drug re-challenge (Figs. 2 and 3).

FACS analysis of the supernatant revealed with *in vitro* PD-L1 blockade the secretion of IL-5, IL-13, and IFN-γ (*n* = 1) after initial priming at 10 µM SMX-NO, secretion of these cytokines from all donors was not present at this priming concentration under standard conditions. Increased IL-10 secretion was observed with PD-L1 blockade after priming at 10 µM for donor 1. Increased secretion of IFN-γ and IL-10 (*n* = 1) and IL-5 and IL-13 (*n* = 2) was observed after priming at 30 µM SMX-NO with PD-L1 blockade compared to priming under standard conditions (Fig. 3A). At priming conditions of 60 µM SMX-NO, increased secretion of IFN-γ and IL-10 (*n* = 1) and IL-5 and IL-13 (*n* = 2) was observed with PD-L1 blockade compared to priming under standard conditions (Fig. 3A). Under PD-L1 block priming conditions dose-dependent secretion of IL-5, IL-13, and IFN-γ was observed for donor 1 with initial priming concentrations of 10 to 60 µM SMX-NO (Fig. 3B) with increased secretion of IL-5 and IL-13 at 30 to 60 µM priming concentrations when compared to standard priming conditions. The secretion of IL-17A, IL-22, and sFasL was below 5 pg/mL (data not shown). The discovery of a mixed Th1 and Th2 secretory profile is a common feature of drug-responsive T-cells (Naisbitt et al. 2003; Gibson et al. 2014). Secretion of Treg cytokines (i.e. IL-10) by drug-responsive T-cells is much rarer and likely indicates the activation of a separate population of clones. A larger number of donors should be studied in future experiments to obtain a more precise assessment of the profile of cytokines secreted from the drug-specific T-cells.

As outlined in previous studies, at “optimal” priming concentrations (i.e. the highest dose that does not lead to suppression of mitogen-induced proliferation), T-cell priming occurred both in the presence and absence of the ICI (albeit with greater re-challenge responses in the presence of ICI). We investigated whether immune checkpoint blockade can lower the threshold for *in vitro* T-cell priming to compounds at sub-optimal priming concentrations where without immune checkpoint blockade T-cell priming could not occur. After *in vitro* T-cell priming in healthy donors to at a range of concentrations with and without PD-L1 blockade, at optimal priming concentrations of drug (60 µM), T-cell priming occurred to SMX-NO regardless of immune checkpoint blockade, (albeit enhanced under such conditions), which is in line with our previous reports (Hammond et al. 2022b). However, for each donor, it was possible to lower the initial priming concentration (In the range of 1 to 30 µM SMX-NO) to one that was not elicitatory in standard conditions. Only under ICI-treated conditions with the *in vitro* administration of PD-L1 blockade, T-cell priming was observed and was able to evoke a response where cells responded after drug re-challenge by proliferating dose-dependently and secreting cytokines (IFN-γ, IL-5, and IL-13). The mechanistic basis of the enabling (low concentration) or augmentation (higher concentrations) of SMX-NO T-cell responses with ICI is not known but may relate to the activation of low-affinity T-cell receptor T-cells and broadening of the drug-specific T-cell repertoire. This might also explain inter-individual variability in the drug-specific T-cell response. In future experiments, we plan to (i) conduct similar experiments using therapeutic anti-PD-L1 drugs and drugs targeting other immune checkpoints (e.g. CTLA-4, TIM3) and (ii) measure variation in checkpoint receptor expression/activity in patients receiving drug treatments.

This work offered a possible explanation as to why ICI patients may be more susceptible to hypersensitivity reactions to their concomitant medications, which may have been tolerated without immune checkpoint blockade. We were able to demonstrate how drug concentrations for T-cell priming vary between individuals. This inter-individual variability offers another explanation as to why some and not all patients become more susceptible to drug hypersensitivity reactions to co-administered agents after ICI.

These observations are significant in that these assays are performed at more physiologically relevant concentrations, and further deconvolute the clinical observations of greater hypersensitivity rates in the relevant patient populations. Previously, we have proposed a hypothetical ICI perturbed hypersensitivity dose-response threshold model (Fig. 4). The role of antigen dose in hypersensitivity reactions is complex and has been an issue of debate for the field. This is because such reactions are multifaceted and depend on numerous factors. What is obvious is that a “necessary but not sufficient” model applies, particularly at first exposure, and that the relevant range is both biphasic (as seen with desensitization procedures) and subject to significant intra and interindividual differences. In a setting where immunoregulatory pathways are inhibited in an individual, a reduction in the dose threshold which is “necessary but not sufficient” for preponderance of aberrant T-cell responses to a drug may be observed. Where the immunomodulatory treatment reduces this threshold enough for it to occur at or below the therapeutic dose level administered to the individual, the individual may experience an adverse reaction (Hammond et al. 2022b). While susceptibilities will vary due to other factors at the level of the individual, the leftward shift of this threshold across a population may equate to a greater number of susceptible individuals at an epidemiological level, which may form reasoning for the recurring observations of increased incidence of hypersensitivity reactions in individuals treated with immune oncology drugs (Hammond et al. 2022a). It is interesting that intraindividual differences are seen within these *in vitro* priming assays, which may signal that at least some of the key components that determine intra-individual susceptibility are recapitulated to an extent. Further work is needed to enhance our understanding of these complex reactions including determining whether other small molecular weight drugs behave in the same manner. This work aids in the development of strategies that can help reduce the incidence of drug hypersensitivity reactions, particularly those that occur due to reduced threshold for T-cell to concomitant medications after checkpoint blockade. By utilizing *in vitro* diagnostic testing, patients that are more susceptible to these reactions can be identified, and specific concomitant medications that are more susceptible to cause these reactions can be replaced or desensitized. This identification can be crucial in allowing patients to continue their immune checkpoint therapy for successful malignant control which may have been ceased due to severe hypersensitivity reactions otherwise.

**Fig. 4.**
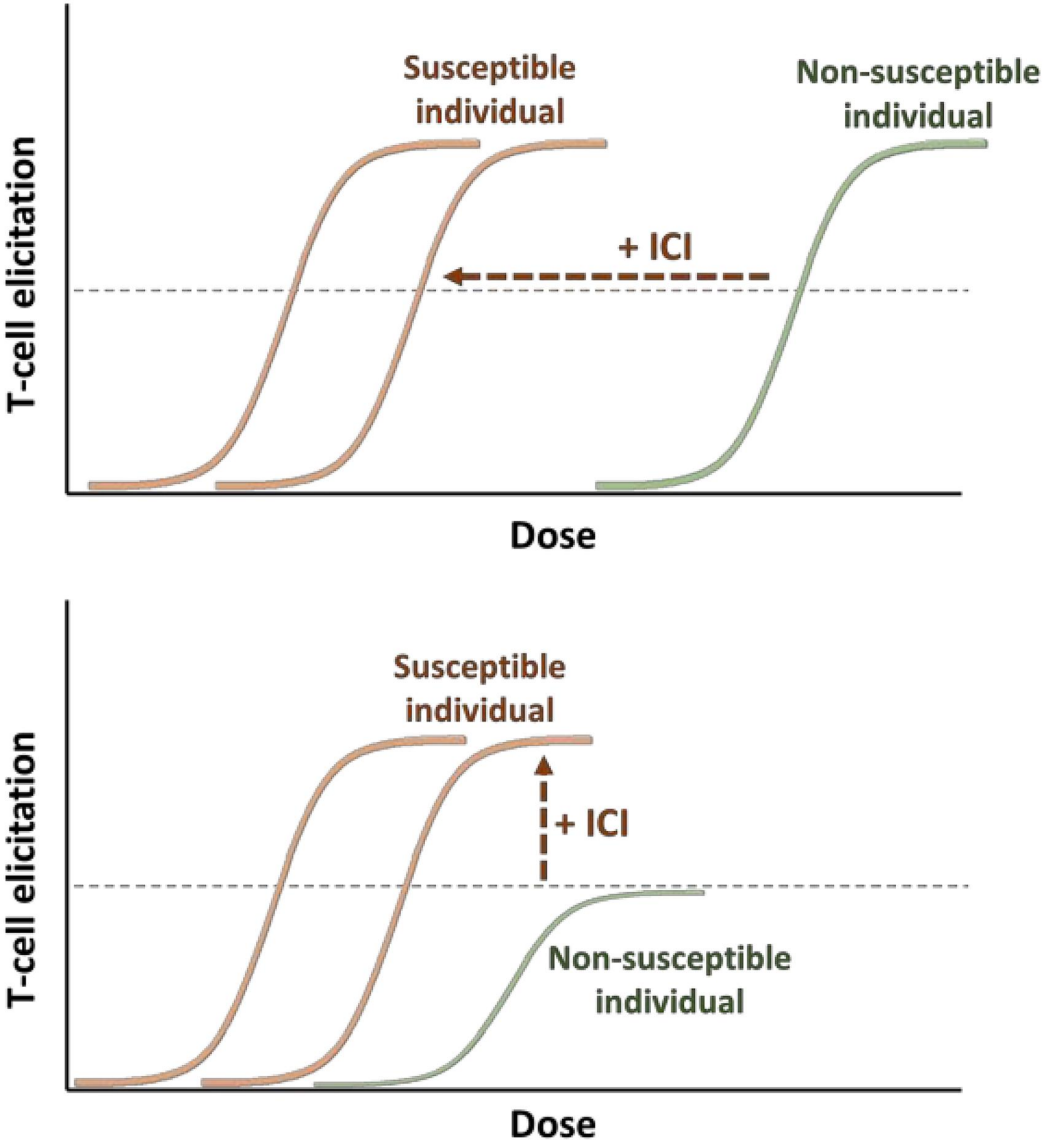
Model for dose dependency of drug hypersensitivity reactions in susceptible and nonsusceptible individuals, and how the addition of immune checkpoint inhibition (ICI) may result in a leftward or upward shift in the dose-response curve, resulting in increased likelihood of T-cell mediated reactions.
